# Quality of Data Entry Using Single Entry, Double Entry and Automated Forms Processing–An Example Based on a Study of Patient-Reported Outcomes

**DOI:** 10.1371/journal.pone.0035087

**Published:** 2012-04-06

**Authors:** Aksel Paulsen, Søren Overgaard, Jens Martin Lauritsen

**Affiliations:** 1 Department of Orthopaedic Surgery and Traumatology, Odense University Hospital, Odense, Funen, Denmark; 2 Institute of Clinical Research, University of Southern Denmark, Odense, Funen, Denmark; 3 Institute of Public Health, Department of Biostatistics, University of Southern Denmark, Odense, Funen, Denmark; Faculty of Health Sciences, University of Cape Town, South Africa

## Abstract

**Background:**

The clinical and scientific usage of patient-reported outcome measures is increasing in the health services. Often paper forms are used. Manual double entry of data is defined as the definitive gold standard for transferring data to an electronic format, but the process is laborious. Automated forms processing may be an alternative, but further validation is warranted.

**Methods:**

200 patients were randomly selected from a cohort of 5777 patients who had previously answered two different questionnaires. The questionnaires were scanned using an automated forms processing technique, as well as processed by single and double manual data entry, using the EpiData Entry data entry program. The main outcome measure was the proportion of correctly entered numbers at question, form and study level.

**Results:**

Manual double-key data entry (error proportion per 1000 fields = 0.046 (95% CI: 0.001–0.258)) performed better than single-key data entry (error proportion per 1000 fields = 0.370 (95% CI: 0.160–0.729), (p = 0.020)). There was no statistical difference between Optical Mark Recognition (error proportion per 1000 fields = 0.046 (95% CI: 0.001–0.258)) and double-key data entry (p = 1.000). With the Intelligent Character Recognition method, there was no statistical difference compared to single-key data entry (error proportion per 1000 fields = 6.734 (95% CI: 0.817–24.113), (p = 0.656)), as well as double-key data entry (error proportion per 1000 fields = 3.367 (95% CI: 0.085–18.616)), (p = 0.319)).

**Conclusions:**

Automated forms processing is a valid alternative to double manual data entry for highly structured forms containing only check boxes, numerical codes and no dates. Automated forms processing can be superior to single manual data entry through a data entry program, depending on the method chosen.

## Introduction

Information in the medical services is now almost exclusively based on electronic recording systems in Denmark, including communication between primary and secondary health care systems [1]. In surgery, among other areas of the health services, there has been a growing focus from medical clinicians on the use of patient-reported outcomes in studies [2]. Internationally, the US Food and Drug Administration has strongly recommended inclusion of patient-recorded outcomes in clinical trials assessing the effect of medical procedures or pharmaceuticals. This has led to a demand for recording larger volumes of information, which traditionally have been collected on paper forms. An alternative to manual data entry has been the introduction of automated reading of such data forms. With an increased focus on measuring and validating measurement tools [3], it is imperative to assess the quality of automated forms processing and this was the motivation for the current study.

In the 1960s, research began on document processing [4]–[5]. With the development of computers and the increasing need to capture large volumes of data, automatic text segmentation and discrimination research gained momentum in the early 1980s [6], [7], [8]. A variety of data processing systems have been described [9], [10], [11], [12], [13], [14], [15], among these different kinds of automatic forms processing or scanning procedures [16], [17], [18]. A growing commercial industry offers automated forms processing technologies and services. However, manual double entry of data is still defined as the definitive gold standard of good clinical practice [19] for data from collected paper forms, and it has been well-validated [18].

Internet-based applications for collecting questionnaires instead of using paper forms may be the future, but for now, and in particular when dealing with an elderly population, it is known that some patient groups do not respond adequately to an Internet-based application for collecting patient-reported outcome questionnaires [20]. When data is entered directly via the internet connection, validation is a very complex matter. No other source of information exists to verify correctness of the data since data is only recorded once.

Automated forms processing technologies are advocated mainly because of potential data quality improvement and likely time and cost reductions. Manual double-key entering of data by key punching is laborious and can be costly. Transcription of data from paper forms into an electronic database can be a nontrivial source of error [21]. Both manual key entering and direct text entry may result in a serious reduction in data quality, if the proportion of erroneous entries is large, as seen in some clinical research databases [22], [23].

Automated forms processing is a method by which one can ‘automatically’ capture information entered into data fields by scanning, and converting it into an electronic format. The data is captured from particular zones and stored in an electronic format. This input method can automate data processing by using pre-defined templates and configurations. A template in this case, would be a map of the document, detailing where the data fields are located within the form. Most of the data are recognised automatically using the pre-specified data characteristics, but if the program is uncertain, verification by a human operator is required.

There are different technologies of automated forms processing. Optical Mark Recognition [OMR] is the least expensive solution but can only be used for recognition of check/mark boxes on a form. The more advanced Intelligent Character Recognition [ICR] can be used for recognition of machine-printed and handwritten characters. In this project, we have used ICR to recognise hand-printed characters, and OMR to identify check boxes filled in by hand on printed forms.

There have been few reports on the quality of automated forms processing and usage in medical settings, relatively few data collection systems are well-described with respect to data quality [24], and further research on automated forms processing performance in this setting is therefore warranted.

The aim is therefore to examine and validate an up-to-date automated forms processing system, by comparing paper-based and scanned patient-reported outcome forms with single and double manually entered data.

## Methods

### Ethics

The study was approved by the Danish National Board of Health and the Danish Data Protection Agency (journal number 2008-41-2593). The Science Ethics Committee of the Region of Southern Denmark rejected registration since this is a registry based study without collection of biological data. The study was carried out in accordance with the World Medical Association’s Declaration of Helsinki, and all patients gave informed written consent to participate. None of the authors have existing or potential competing interests.

**Figure 1:**
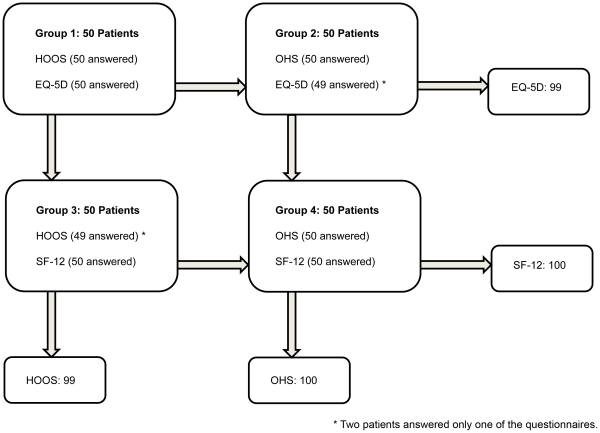
Questionnaire Pairs (200 patients).

### Design

The study was based on a larger study with a cohort of 5777 patients from the Danish Hip Arthroplasty Registry [25]. The cohort consisted of patients over 18 years of age, with primary total hip arthroplasty, regardless of diagnosis, who underwent an operation1–2, 5–6, and 10–11 years earlier. Every patient had received two different patient-reported outcome questionnaires, one general and one disease-specific. The following questionnaires were included in the study: the EuroQoL-5D-3L [EQ-5D], (consisting of the EQ-VAS and the EQ-5D Index) [26], [27], SF-12 Health Survey [SF-12] (yielding MCS and PCS) [28], Hip dysfunction and Osteoarthritis Outcome Score [HOOS] (consisting of HOOS Pain, HOOS PS, and HOOS QoL) [29], and Oxford 12-item Hip Score [OHS] [30]. From the total cohort 200 patients were randomly selected in four groups (blocks) of 50 patients for each year. None of the groups received the same pair of questionnaires so as to maximise the potential for statistical comparison, see Figure 1. Patient characteristics are listed in Table 1. We used paper forms to administer our questionnaires, and postal administration to deliver them.

Sample size and power calculations: Based on 297 available EQ-VAS items, the comparison of methods, assuming an error proportion of 1% by double-entry, had 80% power to detect a 4% higher error proportion by ICR.

**Table 1:** Patient characteristics.

Category	All patients	Group 1	Group 2	Group 3	Group 4
Population (n)	n = 200	n = 50	n = 50	n = 50	n = 50
Percent of total	100	25	25	25	25
Age * (median)	72	72	71	70	74
Range (years)	25–95	47–90	25–90	44–95	34–94
Sex: Female (%)	N = 118 (59%)	N = 29 (58%)	N = 29 (58%)	N = 32 (64%)	N = 28 (56%)

* Age of patients on date of questionnaire dispatch in years.

### Items and Forms

A form was defined as a questionnaire, an item as a single question on a questionnaire and a data field as a possible answer category for an item. EQ-5D contains 6 items in total, 5 single items plus the EQ-VAS. OHS and SF-12 each contains 12 items, and HOOS contains 19 items. The EQ-5D instruction states “By placing a tick in one box in each of the five groups below, please indicate which statements best describe your own health state today”. There are three possible answers (and thus three check boxes resulting in three data fields the scanner is coded to read) for each EQ-5D single item, e.g. for the item ‘Mobility’ categories are: “I have no problems in walking about”, “I have some problems in walking about” and “I am confined to bed”. EQ-VAS requires the respondent to indicate on a thermometer scale from 0 (‘worst imaginable’) to 100 (‘best imaginable’), how good or bad the responder’s health is on that particular day by drawing a line from a box to the appropriate point on the scale which indicates how good or bad his/her health state is on the day. All items from OHS, HOOS, SF-12 and the 5 items on EQ-5D, could be read from the checkboxes by OMR, and only the EQ-VAS had to be read by ICR. The latter was done from a field where the respondent wrote the scale value from 0–100.

Scanning setup was an up-to-date automated forms processing system. The scanner was a Kodak i640 scanner (Kodak Canada Inc., Toronto, Ontario), scanning in 200 DPI, at a speed of 83 pages per minute. Scanning was done in TIFF format, which is approved in Danish law. OCR for AnyDoc ®, version 5.012e (AnyDoc Software Inc., Tampa, Florida) was used for questionnaire setup, and processing. For verifying, AnyDoc®VERIFYIt version 5.0 (AnyDoc Software Inc., Tampa, Florida) was used. HP Elitebook 8530p computers (Hewlett-Packard Company, Palo Alta, California), with Windows version XP and the Microsoft 2003 packages (Microsoft Corporation, Redmond, Washington) were used. Before the study started, we performed template testing of the questionnaires to make sure the setup of the template and placement of the data fields were optimal. The scanner was regularly calibrated. Prior to the scanning, extensive manuals with decision rules for all questionnaires, as well as codebooks were produced to account for any uncertainty. Standard format layout was taken from each questionnaire included with minimal layout adjustments to optimise automated forms processing readability.

### Manual Validation During Scanning

Manual validation was conducted when the automated forms processing system could not convert an answer due to poor or ambiguous questionnaire completion. In these circumstances the scanner stops, and cannot scan further until a human operator manually validates the correct code for the questionnaire answer in question.

### Manual Data Entry

A combined structured questionnaire for all the forms used, including limiting definitions for entry of out of range values was defined using EpiData Data Entry software (EpiData Association, Http://www.epidata.dk). EpiData Entry was also used for the double-key data entry and the program control of the data entry.

### Comparison

To compare the results from automated forms processing with single- and double-key punching, the data were compared using all three methods with EpiData Entry by direct comparisons. The data were also checked for missing values, invalid values and out of range values in STATA, and by reference to the original questionnaires. All manual validations were recorded. A correct data entry was defined when the automated forms processing, single-key punching and double-key punching gave the same data at field (variable) level. In case of differences, we manually consulted the original questionnaire twice, and found the responder’s answer in accordance with the manuals for handling the questionnaires, as well as the individual coding guidance books.

**Table 2:** Number of Questionnaires, Items and Data fields in relation to Processing Method and Questionnaires.

Questionnaire/Scanning method	Total number of Questionnaires	Total number of Items	Total number of Data fields
EQ-5D	99	594	1782
SF-12	100	1200	4700
HOOS	99	1881	9405
OHS	100	1200	6000
Total	398	4875	21887
Scanned with ICR	99	99 *	297 †

* 1 per EQ-5D questionnaire.

† Up to 3 digits per item.

### Statistical Methods

We studied the error proportion overall, for each of the four different questionnaires, and for each individual patient and tabulated this in subgroups by sex and age groups (<60 years, and >60 years) with binomial confidence intervals. Group difference was tested with a Chi Square test. Error proportions were calculated as proportion of errors per 1000 data field with binomial exact 95% confidence intervals (95% CI) (STATA procedure cii). Validation of the automated forms processing in relation to person ID, was done in comparison with the original sample of all patients (n = 5777), with STATA assert command. Descriptive statistics were used to describe patient characteristics. The STATA software Version 10.1 and 11.0 (StataCorp LP, Texas, USA) were used for all statistical analyses. Due to the pre-specified and low number of tests, we saw no reason to adjust the p-level by multicomparison principles.

## Results

The numbers of questionnaires, items and data fields are listed in Table 2. For ICR (Table 3) there was no statistically significant difference between double-key entering (error proportion per 1000 fields = 3.367 (95% CI: 0.085–18.616)) and single-key entering (error proportion per 1000 fields = 6.734 (95% CI: 0.817–24.113), (p = 0.565)), no statistical difference between automated forms processing (error proportion per 1000 fields = 10.101 (95% CI: 2.088–29.234)) and double-key entering (p = 0.319), nor any statistical difference between automated forms processing and single-key entering (p = 0.656). For OMR (Table 4), automated forms processing (error proportion per 1000 fields = 0.046 (95% CI: 0.001–0.258)) performed better than single-key entering (error proportion per 1000 fields = 0.370 (95% CI: 0.160–0.729), (p = 0.020)), double-key entering (error proportion per 1000 fields = 0.046 (95% CI: 0.001–0.258)) performed better than single-key entering (p = 0.020), and automated forms processing and double-key entering performed equally (p = 1.000).

**Table 3:** Errors using Intelligent Character Recognition (ICR).

Category	Single-key entered data	Double-key entered data	Automated forms processing
Number of errors	2 errors	1 error	3 errors
Errors/Questionnaire (n = 99)	2.02% (0.25–7.11)	1.01% (0.03–5.50)	3.03% (0.63–8.60)
Errors/Item (n = 99)	2.02% (0.25–7.11)	1.01% (0.03–5.50)	3.03% (0.63–8.60)
Errors/Data field (n = 297)	0.67% (0.08–2.41)	0.34% (0.01–1.86)	1.01% (0.21–2.92)
Errors/10000 Data fields	67.34	33.67	101.01

n = number scanned, (95% Binomial Confidence Interval).

**Table 4:** Errors using Optic mark Recognition (OMR).

Category	Single-key entered data	Double-key entered data	Automated forms processing
Number of errors	8 errors	1 error	1 error
Errors/Questionnaire (n = 398)	2.01% (0.87–3.92)	0.25% (0.01–1.39)	0.25% (0.01–1.39)
Errors/Item (n = 4776)	0.17% (0.07–0.33)	0.02% (0.00–0.12)	0.02% (0.00–0.12)
Errors/Data field (n = 21608)	0.04% (0.02–0.07)	0.00% (0.00–0.03)	0.00% (0.00–0.03)
Errors/10000 Data fields	3.70	0.46	0.46

n = number scanned, (95% Binomial Confidence Interval).

We found no difference in performance for the different questionnaires with the automated forms processing in OMR (p = 0.609), with double-key entering (p = 0.644), or single-key entering (p = 0.148). Concerning gender, we found no statistical differences for ICR (p = 0.304, p = 0.239, p = 0.095), or OMR (p = 0.409, p = 0.409, p = 0.371). Similarly, there were no differences concerning age for ICR (p = 0.520, p = 0.711, p = 0.711), or OMR (p = 0.687, p = 0.687, p = 0.904).

There were substantial differences in the percentage of manually validated items between the questionnaires and automated forms processing methods: 0.25% (OHS), 0.41% (HOOS Pain), 0.51% (HOOS QoL), 0.61% (HOOS PS), 1.42% (SF-12 PCS and MCS), 2.22% (EQ-5D Index) and 20.20% (EQ-VAS). These differences were statistically significant (p<0.001).

## Discussion

### Summary

We found an extremely low level of error with automated forms processing using OMR. It performed the same as double-key entering and performed better than single-key entering. We found an error level of 0.46 per 10,000 data fields read (OMR), which is better than earlier reports [31].

Concerning ICR, we found an error level per data field of up to one percent in single-key entered data, double-key entered data, and automated forms processing. Only one item (EQ-VAS) required ICR, and therefore relatively few data fields could be included in the ICR analyses, which must be taken into consideration in the interpretation. A very high proportion of items required manual validation on EQ-VAS compared with the other questionnaires, and we will argue that this is because of ICR. It is clearly more difficult for the AFP system to identify a hand-printed character (number) correctly than to identify if a check box is marked, also suggested by the higher number of errors per 10000 data fields in ICR compared to OMR. There are many different ICR systems available, and we cannot rule out that a different ICR system might give better results. Further improvements in ICR technology could possibly decrease the error level to the level of OMR, but this has to be examined in future studies.

### Challenges with Data Quality of Questionnaires

There are many potential errors from questionnaire data. The table from the work of Reider and Lauritsen [32] conceptualizes the potential errors arising from data capture, poor design of the data entry form, no program constraints on data entry, single-entry manual key punching and lack of validation in these studies. Automated forms processing has the potential to remove some of these pitfalls, and potentially improve data quality from questionnaires. Relying on internet based data entry could result in an error level comparable to single manual data entry, but the validity of internet based solutions warrants further research in particular in relation to possible age and or subgroup differences potentially resulting in information bias.

Cost: Further studies should assess the cost of modern automated forms processing systems. Earlier reports have shown processing time was reduced to about one half to one third of that of manual data entry and wage expenses were reduced to about one third to one quarter, but found that a very large number of forms needed to be processed in order to recover the considerable initial investment [31]. Even though the cost of equipment for automated forms processing data capture has decreased considerably in recent decades, substantial time and computer expertise is still required for implementation.

We believe our study is representative of a wide variety of research and clinical settings where paper form questionnaires are used. Total hip arthroplasty is indicated for patients with pain and functional disabilities or reduced quality of life. The population is an extensively studied elderly population, with a mean age in Denmark of 70/67 years (female/male), the patients have a spectrum of comorbid conditions and they constitute a suitable and interesting population in relation to validation of automated forms processing.

### Benefits

There are several potential benefits of using automated forms processing, including a low error level, an improved data verification process and, more importantly, (especially in big studies with many respondents) a significant reduction in time required for data entry [33]. In a registry setting, it is important to achieve an efficient data collection procedure. Some studies find automated forms processing approximately three times as fast as the standard method of data entry, with a digit recognition rate of 92.4% [34]. The quality of automated forms processing has been found in earlier reports to be acceptable, and studies report a data entry error of as low as 0.041% for all questionnaire items [35]. Automated forms processing was validated in Denmark in 1998, and was then found to perform slightly better than single data entry, but worse than double data entry [31]. However, in the last 12 years, there has been a rapid development in both software and hardware, and we have found that an up-to-date system can perform as well as double-key manual entry.

### Conclusion

Automated forms processing can yield excellent results provided use of highly structured questionnaires. OMR performed equally as well as manual double-key entering, and better than single-key entering. Regarding ICR, we cannot draw firm conclusions due to the limited data available in this study, and therefore further research, as well as improvement in ICR technology, is warranted.
